# Changes in anorectal physiology following injection sclerotherapy using aluminum potassium sulfate and tannic acid versus transanal repair in patients with symptomatic rectocele; a retrospective cohort study

**DOI:** 10.1186/s12893-018-0363-x

**Published:** 2018-05-31

**Authors:** Joo Hyung Kim, Yong Pyo Lee, Kwang Wook Suh

**Affiliations:** 1 Department of Surgery, Ajou University School of Medicine, 164 World cup-ro, Yeongtong-gu, Suwon, Gyeonggi-do 16499 Republic of Korea; 2 Department of Surgery, Hanvit Hospital, 1017 Gyeongsu-daero, Jangan-gu, Suwon, Gyeonggi-do 16300 Republic of Korea

**Keywords:** Rectocele, ALTA (aluminum potassium sulfate and tannic acid), Injection sclerotherapy, Transanal rectocele repair

## Abstract

**Background:**

Following injection sclerotherapy using ALTA (aluminum potassium sulfate and tannic acid) (ALTAS) and transanal rectocele repair (TAR), changes in anorectal physiology were analyzed to compare the significance of the two treatments.

**Methods:**

ALTAS was administered to 23 patients and 18 patients were treated using TAR. Efficacy measures included changes in defecography, anorectal manometry and constipation scoring system value.

**Results:**

This was a retrospective cohort analysis conducted on prospectively collected data. Comparing anorectal physiology pre- and post- ALTAS, a statistically significant difference in push was observed with pre-ALTAS treatment (pre-A) at 104.33 ± 4.91° compared with post-ALTAS treatment (post-A) at 113.95 ± 4.74° (*p* < 0.001). With a pre-A value of 1.55 ± 0.18 cm and a post-A value of 2.46 ± 0.34 cm, perineal descent also showed an increase as well (*p* < 0.001). The rectocele size decreased post-A from a pre-A value of 7.74 ± 0.86 cm compared with a post-A value of 2.91 ± 0.52 cm (*p* < 0.001). The rectal sensation improved post-A compared with pre-A. Comparing anorectal physiology results of ALTAS and TAR treatments, no differences in defecography and rectal sensation were detected pre- and post-treatment. However, in terms of anorectal manometry, the mean resting pressure and maximal squeezing pressure showed statistical difference with two treatments.

**Conclusions:**

ALTAS treatment is a feasible option resulting in rapid and effortless long-term outcome, with low rates of complications. Therefore, this treatment may be an effective alternative for patients with symptomatic rectocele.

## Background

Several patients with chronic constipation require medical treatment. However, patients with rectocele can easily be treated surgically [1, 2], suggesting that research investigations may yield a significant outcome. Rectocele is a disease, in which the anterior wall of the lower rectum and posterior vaginal wall are curved and protrude into vaginal canal due to defective or weak rectovaginal septum [3]. Enlarged rectocele and functional disability may result in retention of the feces in the rectum. In this case, insertion of finger into the vaginal wall may facilitate the excretion of the feces by pushing them down the rectum in some patients. Rectocele was found in 20–81% of normal female population [4–7], and 23–70% of patients with rectocele showed symptoms such as constipation, suggesting unclear relationship between this anatomical disorder and symptoms of rectocele [8, 9]. Current treatment methods for rectocele include transanal rectocele repair (TAR) [10–12], posterior colporrharphy [13], transvaginal repair using Marlex mesh [14], and laparoscopic transabdominal approach [15]. However, physicians select the treatment method based on their familiarity with specific procedures [16] in the absence of robust evidence supporting individual treatments or comparative analysis of different treatments. Recently, injection sclerotherapy using a combination of aluminum potassium sulfate and tannic acid (ALTA) has been reported as a possible treatment for rectocele because of its simplicity, low percentage of complications, and lack of apparent signs of pain [17]. Unfortunately, studies investigating anal physiology are lacking.

In this study, patients with rectocele were divided into two groups: TAR and sclerotherapy using ALTA (ALTAS). Following these treatments, changes in anorectal function were analyzed using anorectal manometry and defecography to obtain comparative results and determine the significance of ALTAS.

## Methods

### Patients

Between January 2010 and October 2013, patients from Hanvit hospital coloproctology clinic who listed chronic constipation as their chief complaint were reviewed to determine the presence of rectocele based on anal ultrasonography, anorectal manometry, and defecography. A total of 48 patients were subjected to ALTAS and 32 patients underwent TAR. Three months after these surgeries, patients were followed up with anorectal physiological studies. Three years post-treatment, a total of 23 patients who underwent ALTAS and 18 patients who were treated with TAR consented to participate. This was a retrospective cohort analysis conducted on prospectively collected data. This study was approved by the Institutional Review Board of Ajou University Hospital, Republic of Korea (No. AJIRB-MED-MDB-17-493). The Institutional Review Board exempted the requirement for informed consent because we assessed on de-identified data retrospectively.

### Outcome measurements

A validated scoring system (constipation scoring system [CSS]) was used to assess the severity of constipation [6]. The scoring system includes eight categories and the scores ranged from 0 (normal) to 30 (severe constipation). All patients underwent complete proctological examination, followed by anal ultrasonography, anorectal manometry and defecography. During defecography, the anorectal angle, perineal descent, and size of rectocele during resting, squeezing, and push were reviewed.

Anorectal angle was defined as the angle between the anal canal and the tangential line. The tangential line extended from the posterior rectal wall right below the impression, which was immediately adjacent to the upper anal canal. Based on the perineal descent (resting value minus defecation value), movement across the anorectal junction during ‘squeeze’ and defecation were calculated. The size of rectocele was calculated as the maximum depth of the bulge beyond the hypothesized and conventional line of the anterior rectal wall [18].

Patients were expected to fulfill at least two out of three conditions, which include constipation, for a diagnosis of rectocele indicated for surgery. The first condition was the size of rectocele greater than 3 cm. The second condition was retention of barium contrast in the anus even after more than three attempts of defecation, post-defecography. The final condition was application of rectal pressure via insertion of finger in the vagina to facilitate defecation [7, 18]. Postoperatively, 3 months after surgery, patients underwent defecography and anorectal manometry similar to pre-surgical intervention. The anal functionality used in this study was based on methods used by Nguyen and Lubowski [19] and Ger et al. [20].

**Fig. 1:**
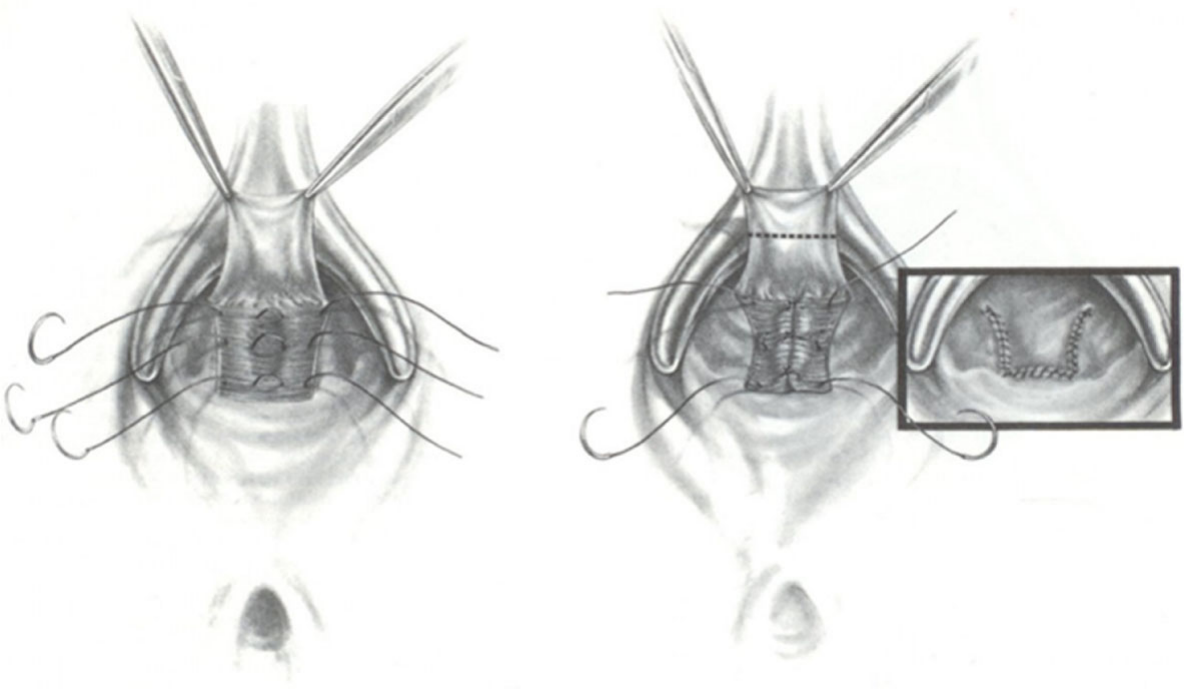
Transanal approach of rectocele repair (TAR). This approach aimed to reduce anterior rectal capacity by placating the full thickness of the anterior rectal wall up to 5–7 cm into the rectum

TAR was performed as an operation for rectocele in a jack-knife position. Along the dentate line, a transverse incision was made. Via two vertical lacerations at either end, which extended for about 7 cm. Until the area above the weakness in the septum was converged, a muco-muscular flap was raised with a wider base. Starting from the dentate line and developing adjacently, three to four interrupted transverse sutures of 2–0 Vicryl® (Ethicon, Norderstedt, Germany) were used to increase the lax rectovaginal septum without infiltrating the vaginal mucosa. Additional two to three vertical sutures were used between the closest and farthest points, increasing the rectovaginal septum to curtail the anterior rectal wall. Lastly, the edge of the flap was stitched to the dentate line with ongoing 4–0 Vicryl® (Ethicon, Norderstedt, Germany). The lateral lacerations were measured comparatively [10, 11, 21] (Fig. 1).

**Fig. 2:**
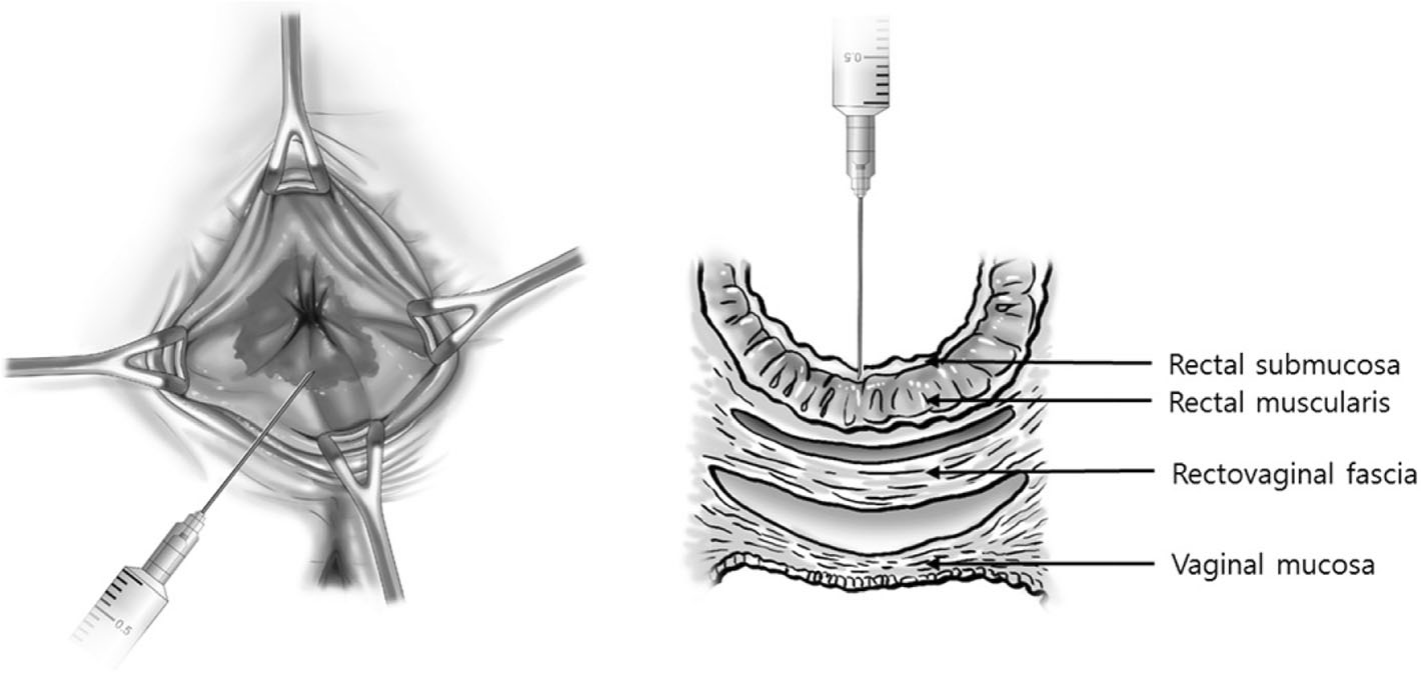
Aluminum potassium sulfate and tannic acid (ALTA) sclerotherapy. The rectum was prolapsed to its maximum extent using forceps. Along the rectal submucosa, 1~ 2 mL of ALTA solution was circumferentially injected using a 5 mL syringe equipped with a 25-gauge injection needle and at 10 to 20 different places at the edge of the rectocele to the dentate line

**Fig. 3:**
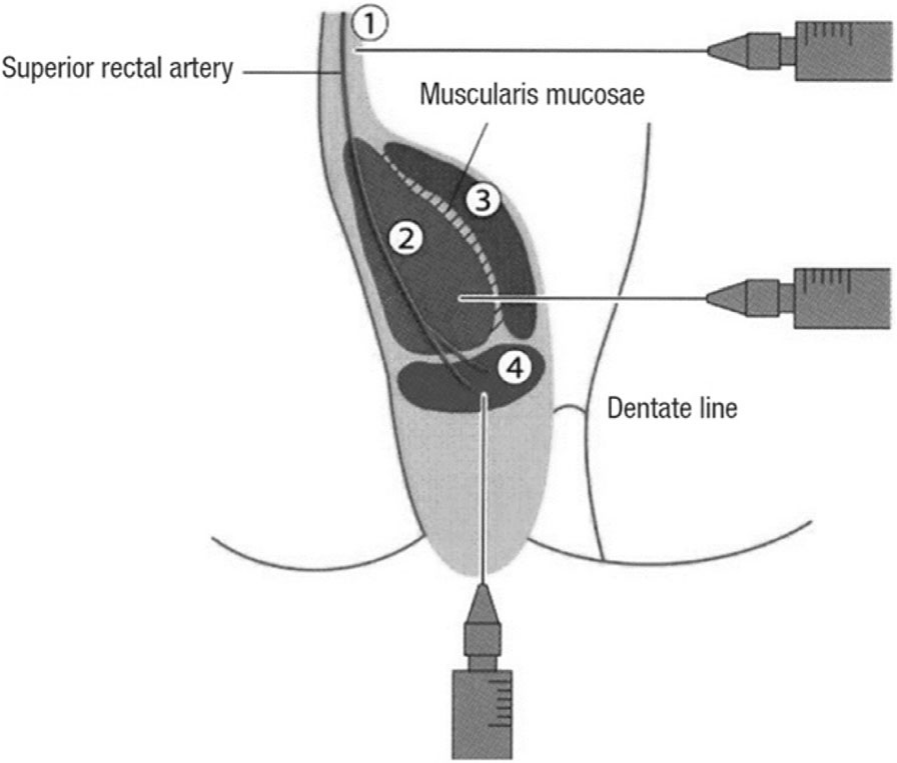
Four-step injection of aluminum potassium sulfate and tannic acid. ① Submucosa at the superior pole of hemorrhoids ② Submucosa in the central part of hemorrhoids ③ Mucous lamina propria in the central part of hemorrhoids ④ Submucosa at the inferior pole of hemorrhoids

By taping the buttocks wide apart, the ALTAS was performed under saddle-block anesthesia in the prone jack-knife position. A glycerin enema was used to empty the rectum pre-operatively. In case of coexisting intussusception, a forceps was used to cascade the redundant rectum. Along the rectal submucosa, 1~ 2 mL of ALTA solution (Ziohn®, Yuhan Medica Corporation, Seoul, Republic of Korea) was injected using a 5 mL syringe equipped with a 25-gauge injection needle and at 10 to 20 different places at the edge of the rectocele [17] (Fig. 2). In terms of coexisting hemorrhoids, the physician used a Z-type proctoscope (Arakawa Seisakujo, Tokyo, Japan) with a small opening. ALTA solution was administered based on a 4-step injection procedure [22, 23] (Fig. 3). After the operation, patients were administered with prophylactic oral antibiotics (cefaclor, 750 mg/day) and oral analgesia (Etodolac, 600 mg/day) for 3 days without any dietary restrictions. Patients were monitored on an outpatient basis using anorectal physiology study and CSS as the primary efficacy measures. Patients were evaluated by the physicians before and 3 months after the treatment. CSS were evaluated 3 years after the treatment by contacting the patients to determine any changes in their symptoms.

### Statistical analyses

All statistical analyses were performed using IBM® Statistics SPSS® for Windows 23.0 (SPSS Inc., Chicago, IL, USA). Variables such as scale value of anorectal physiology tests and CSS of ALTAS and TAR were analyzed by paired-samples t-test. Age and comparative treatment method were analyzed by independent-samples t-test. CSS of baseline, after 3 months, after 3 years were verified through one-way ANOVA. Statistical significance was considered when *P* value was less than 0.05.

## Results

### Age, operative duration, total injection dose of ALTA

The average age of patients who underwent ALTAS was 53.70 ± 7.17 years, and that of patients who were treated with TAR was 51.89 ± 8.37 years (*P* = 0.461). The operative duration for ALTAS was 7.74 ± 2.85 min, and that of TAR was 49.17 ± 11.79 min. The mean total injection dose of ALTA was 27.39 ± 7.62 mL (range,18–45 mL). No postoperative complications were found in any patients.

### CSS comparison of pre-treatment, 3 months and 3 years post-treatment

The baseline value in patients who underwent ALTAS was 11.61 ± 2.84, 3 months post-treatment was 4.78 ± 1.09, and 3 years post-treatment was 5.61 ± 0.94. Comparative analysis of these results showed that the treatment had a statistical significance (*P* < 0.001). The baseline value for patients who underwent TAR was 10.83 ± 2.55, 3 months post-treatment was 4.39 ± 0.61, and 3 years post-treatment was 5.44 ± 1.04, suggesting that TAR also had a statistical significance (*P* < 0.001), and there was no sign of relapse.

There was no difference in CSS between the two treatments in terms of pre-treatment, 3 months post-treatment, and 3 years post-treatment outcomes (Table 1).

**Table 1:** Comparison of constipation scoring system between ALTAS and TAR in rectocele

Treatment	Baseline value^a,b^	After 3 months^a^	After 3 years^b^	*P* value^a,b^
ALTAS	11.61 (2.84)	4.78 (1.09)	5.61 (0.94)	< 0.001
TAR	10.83 (2.55)	4.39 (0.61)	5.44 (1.04)	< 0.001
*P*-value	0.368	0.176	0.600	

Data are expressed as absolute mean (standard deviation)

Minimum Score, 0; Maximum Score, 30

*ALTAS* sclerotherapy using aluminum potassium sulfate and tannic acid, *TAR* transanal rectocele repair

^a^significant data between baseline value and after 3 months

^b^significant data between baseline value and after 3 years

### Comparing anorectal physiology pre-ALTAS treatment (pre-A) and post-ALTAS treatment (post-A)

Defecography results showed that the resting and squeezing performance from the anorectal angle showed no difference between pre-A and post-A. However, there was a statistically significant difference in push with a pre-A) at 104.33 ± 4.91° compared with post-A at 113.95 ± 4.74° (*P* < 0.001). With a pre-A of 1.55 ± 0.18 cm and post-A of 2.46 ± 0.34 cm, perineal descent also showed an increase (*p* < 0.001). The rectocele size decreased post-A from a pre-A of 7.74 ± 0.86 cm to a post-A value of 2.91 ± 0.52 cm (*P* < 0.001).

In anorectal manometry, both mean resting pressure (MRP) and maximal squeeze pressure (MSP) were not different pre-A and post-A. In rectal sensation, sensory threshold (ST) showed a pre-A value of 85.53 ± 11.30 mL and a post-A value of 78.48 ± 11.91 mL (*P* = 0.002). The earliest defecation urge (EDU) was recorded at a pre-A level of 158.71 ± 23.70 mL, and a post-A level of 116.97 ± 25.07 mL (*p* < 0.001). Finally, the maximal tolerable volume (MTV) decreased significantly from a pre-A level of 207.64 ± 34.12 mL to a post-A level of 148.48 ± 22.38 mL (*P* < 0.001) as shown in Table 2.

**Table 2:** Comparison of defecographic and anal manometric findings between pre- and post-treatment of ALTAS in rectocele

ALTAS	Pre-treatment	Post-treatment	*P* value
Defecography, mean (SD)			
Anorectal angle (°)			
Resting	98.86 (5.03)	98.05 (4.24)	0.153
Squeezing	91.30 (0.60)	90.84 (2.50)	0.400
Push	104.33 (4.91)	113.95 (4.74)	< 0.001
Perineal descent (cm)	1.55 (0.18)	2.46 (0.34)	< 0.001
Rectocele size (cm)	7.74 (0.86)	2.91 (0.52)	< 0.001
Anorectal manometry, mean (SD)			
MRP (mmHg)	19.12 (1.98)	19.66 (2.55)	0.394
MSP (mmHg)	86.17 (9.40)	85.57 (9.28)	0.791
Rectal sensation (mL)			
ST	85.53 (11.30)	78.48 (11.91)	0.002
EDU	158.71 (23.70)	116.97 (25.07)	< 0.001
MTV	207.64 (34.12)	148.48 (22.38)	< 0.001

Data are expressed as absolute mean (standard deviation)

*ALTAS* sclerotherapy using aluminum potassium sulfate and tannic acid, *SD* standard deviation, *MRP* mean resting pressure, *MSP* maximal squeezing pressure, *ST* sensory threshold, *EDU* earliest defecation urge, *MTV* maximal tolerable volume

### Comparing anorectal physiology pre-TAR treatment (pre-T) and post-TAR treatment (post-T)

Similar to ALTAS, defecography showed no difference in resting and squeezing phases of anorectal angle pre-T and post-T. However, with pre-T at 106.10 ± 5.94°, post-T 112.21 ± 4.92°, the push phase showed statistically significant difference (*P* < 0.001). Perineal descent of 1.58 ± 0.23 cm occurred with pre-T compared with 2.43 ± 0.38 cm post-T, suggesting a significant increase post-T (*P* < 0.001).

In anorectal manometry, MRP of 20.18 ± 2.90 mmHg occurred at pre-T: compared with 21.93 ± 3.54 mmHg post-T (*p* = 0.049), while MSP was 87.87 ± 11.45 mmHg pre-T: compared with 94.23 ± 11.31 mmHg post-T (*P* < 0.001). These results showed that, unlike ALTAS treatment, there was a significant increase post-T. In rectal sensation, similar to ALTAS, ST showed a pre-T value of 85.67 ± 11.25 mL and a post-T value of 78.06 ± 12.02 mL (*P* = 0.001), while EDU showed a pre-T value of 157.86 ± 22.54 mL compared with post-T level of 109.19 ± 22.20 mL (*P* < 0.001). The MTV value pre-T was 197.52 ± 31.35 mL compared with a post-T value of 142.22 ± 23.83 mL (*P* < 0.001), which were significant statistically (Table 3).

**Table 3:** Comparison of defecographic and anal manometric findings between pre- and post-treatment of TAR in rectocele

TAR	Pre-treatment	Post-treatment	*P* value
Defecography, mean (SD)			
Anorectal angle (°)			
Resting	97.67 (3.78)	99.22 (5.06)	0.229
Squeezing	91.79 (3.65)	91.76 (3.84)	0.979
Push	106.10 (5.94)	112.21 (4.92)	< 0.001
Perineal descent (cm)	1.58 (0.23)	2.43 (0.38)	< 0.001
Rectocele size (cm)	7.78 (1.00)	2.67 (0.69)	< 0.001
Anorectal manometry, mean (SD)			
MRP (mmHg)	20.18 (2.90)	21.93 (3.54)	0.049
MSP (mmHg)	87.87 (11.45)	94.23 (11.31)	< 0.001
Rectal sensation (mL)			
ST	85.67 (11.25)	78.06 (12.02)	0.001
EDU	157.86 (22.54)	109.19 (24.20)	< 0.001
MTV	197.52 (31.35)	142.22 (23.83)	< 0.001

Data are expressed as absolute mean (standard deviation)

*TAR* transanal rectocele repair, *SD* standard deviation, *MRP* mean resting pressure, *MSP* maximal squeezing pressure, *ST* sensory threshold, *EDU* earliest defecation urge, *MTV* maximal tolerable volume

### Comparing anorectal physiology results of ALTAS and TAR treatments

No differences in defecography and anorectal manometry index are found pre-treatment. No differences in defecography and rectal sensation were detected post-treatment. However, in terms of anorectal manometry, the MRP and MSP showed statistical difference with two treatments (MRP: *P* = 0.022, MSP: *P* = 0.010) (Table 4).

**Table 4:** Comparison of anorectal physiology between ALTAS and TAR in rectocele

Anorectal physiologic study	Pre-treatment			Post-treatment		
	ALTAS	TAR	*P* value	ALTAS	TAR	*P* value
Defecography, mean						
Anorectal angle (°)						
Resting	98.86	97.67	0.411	98.05	99.22	0.426
Squeezing	91.30	91.79	0.664	90.84	91.77	0.358
Push	104.33	106.10	0.303	113.95	112.21	0.261
Perineal descent (cm)	1.55	1.58	0.642	2.46	2.43	0.808
Rectocele size (cm)	7.74	7.78	0.895	2.91	2.67	0.196
Anorectal manometry, mean						
MRP (mmHg)	19.12	20.18	0.172	19.66	21.93	0.022
MSP (mmHg)	86.17	87.87	0.605	85.57	94.23	0.010
Rectal sensation (mL)						
ST	85.53	85.67	0.967	78.48	78.06	0.911
EDU	158.71	157.86	0.907	116.97	109.19	0.323
MTV	207.64	197.52	0.335	148.48	142.22	0.397

Data are expressed as absolute mean

*ALTAS* sclerotherapy using aluminum potassium sulfate and tannic acid, *TAR* transanal rectocele repair, *MRP* mean resting pressure, *MSP* maximal squeezing pressure, *ST* sensory threshold, *EDU* earliest defecation urge, *MTV* maximal tolerable volume

## Discussion

Rectocele has always been considered as a gynecologic problem. It was not until 1968 when Sullivan et al. [12] introduced TAR that rectocele was considered as another type of chronic constipation amenable to anorectal surgery. Recent advances in anorectal physiology elucidated the factors underlying the increased size of rectocele and the inability of the rectocele to promote defecation in the direction of the anal canal during defecation. Instead, defecation is diverted towards the weakened rectovaginal septum and additional push is needed for excretion, which enlarges the rectocele [8]. The rectocele can be examined by physical examination digitally using anal finger. However, the significance of rectoceles that are larger than 2 cm is disputed [7, 24], with a few experts suggesting that the size is not correlated with the symptoms [25–27]. These disagreements highlight several controversies floating around regarding the relevance of rectocele size and symptoms. Therefore, several clinicians are uncertain regarding the appropriate timing for surgery and the specific surgical intervention that is most appropriate.

Anatomical and physiologic studies of rectocele are essential to address this challenge. As the CSS level decreased pre-A, post-A and pre-T, post-T, the rectocele size was significantly reduced in defecography. The treatment effect was sustained without any significant differences between the two groups in CSS testing, suggesting that ALTAS is an effective intervention for the management of rectocele. Additionally, in defecography, a significant increase was observed in anorectal angle under exertion. Another significant increase occurred in perineal descent after surgery compared with pre-surgical level. Van Laahoven et al. [2] interpreted that the size of rectocele shifted the vector force of defecation in a physiological direction post-operation. In anorectal manometry, which is used to determine the physiological changes, the MRP indicating the functionality of internal sphincter muscle, and MSP indicating the function of the external sphincter muscle, were significantly lowered postoperatively. This result was attributed to damage of anal muscles due to anal retractor, which is used during TAR. In addition to the effect of anatomical correction, surgery also improved the rectal sensation in both ALTAS and TAR, which significantly lowered the ST, EDU, MTV, and rectal sensation post-surgery.

Currently, treatments such as TAR [10–12], posterior colporrpaphy [13], transperineal repair, which uses Marlex mesh [14], and laparoscopic transabdominal approach [15], stapled transanal rectal resection (STARR) [28] are used to treat rectocele. TAR, which is an operative method used by many coloproctologists, has been used to treat anal diseases such as hemorrhoids, mucosal prolapse, and complete rectal prolapse, simultaneously [29]. However, the disadvantages of TAR related to its inability to treat vaginal disease in addition to increasing the risk of complication associated with vaginal tightness [30]. Posterior colporrhaphy, used by several gynecologists can be used to treat urological or gynecological disease at the same time. However, it cannot be used to treat anal disease, and is associated with extreme postoperative pain and complications of vaginal strictures [13]. Transperineal repair, which uses Marlex mesh, is a new treatment modality that has a theoretical advantage of recovery of anatomical function by attaching the prostatic patch to the anatomically defective area. However, this treatment leads to fibrotic tissue formation and associated pain as well as perineal pain and dysuria [14]. Other disadvantages include prolonged scar healing, increased discharge, and continuous administration of antibiotics [21]. Laparoscopic transabdominal approach is indicated for cases of relapse or when rectal intussusception accompanies rectal prolapse. However, it is a larger procedure compared with other surgeries [15].

Longo came up with STARR for patients diagnosed with obstructed defecation through rectocele and intussusception. While STARR was very effective in improving the rectal symptoms related to rectocele and intussusception, it was associated with complications [28]. The postoperative bleeding rate ranged from 3.3 to 26.6%, and fecal urgency rate ranged from 1.1 to 34%, while incontinence to flatus rate ranged from 6 to 26.7%. The recurrence rate was less than 40% [31, 32].

Injection sclerotherapy is indicated for hemorrhoids and rectal prolapsed. It is minimally invasive and economical, but seldom used to treat rectocele [33]. ALTA treatment interrupts the blood flow to the hemorrhoids resulting in sporadic hemostatic effect and contraction of hemorrhoids. Eventually, sterile inflammation induces persistent fibrosis. Further, attachment and fixation of submucosal layer and mucosa to the muscular layer is promoted. Finally, bleeding of hemorrhoids is stopped and the prolapse is resolved [34].

Abe et al. [17] reported injection sclerotherapy as a treatment that is easy, rapid, and shows fair mid-term results. More importantly, it is associated with a low rate of complications. Another advantage of ALTAS is that it does not need suturing or excision, unlike transanal procedures. The treatment is not associated with the risk of anastomotic dehiscence or bleeding, and can be performed rapidly under local anesthesia, without any postoperative complications such as rectovaginal fistula or infection and alleviates postoperative pain. ALTAS can be used to treat hemorrhoids, rectal intussusception, and mucosal prolapse, which are concomitant anorectal diseases, simultaneously. ALTAS, using the Z-type proctoscope, provides effective access to high rectoceles as well [17].

Complex internal muscle control such as nerve plexuses, muscle, colon and pelvic floor activity and various chemical and hormones play an important role in normal defecation [35]. Therefore, psychologic factors, long-term idiopathic constipation, and hormonal disorders can have a huge impact on defecation impairment [2]. Rectocele, which is one of the main causes of defecation disability, is an anatomical abnormality suggesting that surgery that reduces the size of the rectocele effectively improves the symptoms. However, even though the size of the rectocele is reduced after the surgery, it does not completely disappear, which suggests that the relationship between postoperative anatomical correction and symptom improvement is still unclear [36]. Our study has shown that both treatments significantly improved the rectal sensitivity, consistent with Van Laarhoven et al. [2]’s findings suggesting that the significantly reduced size of rectocele post-treatment alters the vector force in the normal, physiological direction. However, the concept of physiological changes in improving rectal sensation along with the underlying mechanism needs to be explored further.

## Conclusions

Both ALTAS and TAR effectively reduced the size of rectocele, which resulted in alleviation of symptoms such as constipation. It is believed that these two treatments had additional effects beyond reducing the size of rectocele. ALTAS and TAR treatments appear to show synergistic effects such as alleviation of rectal sensation and altering the push phase of the bowel movement in the physiological direction. Additionally, ALTAS treatment was a feasible option for effortless and rapid as well as long-term outcome, with low rates of complications, and it represents a substitute for patients with symptomatic rectocele.

## Abbreviations

ALTA: Aluminum potassium sulfate and tannic acid
ALTAS: Sclerotherapy using aluminum potassium sulfate and tannic acid
CSS: Constipation scoring system
EDU: Earliest defecation urge
MRP: Mean resting pressure
MSP: Maximal squeezing pressure
MTV: Maximal tolerable volume
post-A: Post-sclerotherapy using aluminum potassium sulfate and tannic acid treatment
post-T: Post-transanal rectocele repair treatment
pre-A: Pre-sclerotherapy using aluminum potassium sulfate and tannic acid treatment
pre-T: Pre-transanal rectocele repair treatment
ST: Sensory threshold
STARR: Stapled transanal rectal resection
TAR: Transanal rectocele repair
